# Metal-free C–H mercaptalization of benzothiazoles and benzoxazoles using 1,3-propanedithiol as thiol source

**DOI:** 10.3762/bjoc.15.24

**Published:** 2019-01-29

**Authors:** Yan Xiao, Bing Jing, Xiaoxia Liu, Hongyu Xue, Yajun Liu

**Affiliations:** 1 School of Pharmaceutical Engineering, Shenyang Pharmaceutical University, 103# Wenhua Road, Shenyang, Liaoning 10016, China; 2 School of Life Science and Medicine, Dalian University of Technology, 2# Dagong Road, Panjin, Liaoning 124221, China

**Keywords:** benzothiazole, benzoxazole, C–H functionalization, mercaptalization, 1,3-propanedithiol

## Abstract

A facile and effective C–H functionalization strategy for the synthesis of 2-mercaptobenzothiazoles and 2-mercaptobenzoxazoles is described. 1,3-Propanedithiol was employed to convert benzothiazoles and benzoxazoles to the corresponding heteroarylthiols in the presence of potassium hydroxide and DMSO. This novel protocol is featured by direct C–H mercaptalization of heteroarenes and a simple reaction system.

## Introduction

**Figure 1:**
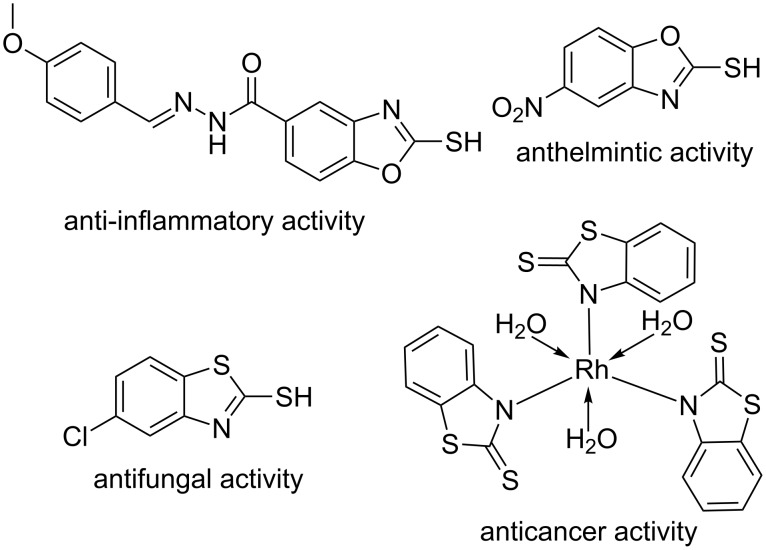
Representative examples of biologically active 2-mercaptobenzoxazoles and 2-mercaptobenzothiazoles.

**Scheme 1:**
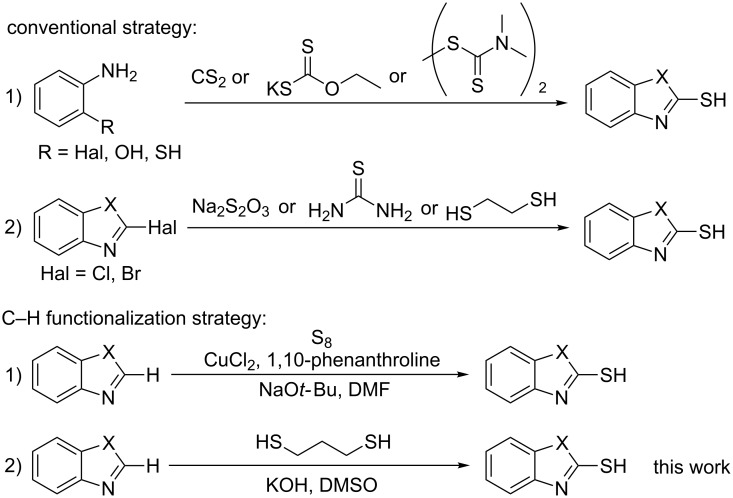
Strategies for the synthesis of 2-mercaptobenzothiazole and 2-mercaptobenzoxazole (X = O, S).

Both 2-mercaptobenzothiazoles and 2-mercaptobenzoxazoles are not only fundamental building blocks in organic synthesis, but also possess various biological activities (Figure 1) [1–2]. A complex of a transition metal (such as Ru, Pt, Bi, etc.) with either a 2-mercaptobenzoxazole or a 2-mercaptobenzothiazole often provides cytotoxic activity against cancer cells [3–5]. 2-Mercapto-*N*-(substituted arylidine)benzoxazole-5-carbohydrazide derivatives have promising anti-inflammatory activities [6]. 2-Mercapto-5-nitro-1,3-benzoxazole and its derivatives shows strong anthelmintic activity [7]. 2-Mercapto-5-chloro-1,3-benzothiazoles possess antifungal activity against *Candida albicans* and *Candida tropicalis* [8] and 2-mercapto-1,3-benzothiol and its derivatives exhibit inhibitory effects against thyroid peroxidase [9].

(Hetero)aryl thiols are often prepared from the corresponding halides through direct nucleophilic substitution [10–12] or metal-catalyzed C–S coupling reactions [13]. Conventional methods for the synthesis of 2-mercaptobenzoxazoles and 2-mercaptobenzothiazoles include the interaction of 2-aminophenol or 2-haloanilines with carbon disulfide [14–16], or potassium ethyl xanthate [17–18] (Scheme 1). In 2017, the Dong group reported a new method for the synthesis of 2-mercaptobenzoxazoles and 2-mercaptobenzothiazoles by cyclization of 2-aminothiophenols or 2-aminophenols with tetramethylthiuram disulfide in water [19]. Very recently, the Liu group developed a novel protocol for the synthesis of 2-mercaptobenzothiazoles via a three-component reaction of *o*-iodoanilines and K_2_S in DMSO [20]. Another way to prepare 2-mercaptobenzothiazoles and 2-mercaptobenzoxazoles is the nucleophilic substitution of 2-halo-substituted benzothiazoles and benzoxazoles with sulfur-containing reagents including sodium thiosulfate [21], thiourea [22] and 1,2-ethanedithiol [23].

In the past decades, C–H functionalization has become an effective strategy for constructing different molecules directly from simple arenes and alkanes. C–H functionalization is an important method for C–S coupling reactions [24–25]. For example, transition metal-catalyzed C–H thiolation of benzothiazole or benzoxazole with a disulfide and a thiol provides easy access to the corresponding sulfides [26–34]. However, the examples using C–H functionalization for preparing 2-mercaptobenzoxazoles or 2-mercaptobenzothiazoles are still rare. In 2009, the Daugulis group reported that benzoxazole was converted to 2-mercaptobenzoxazoles in the presence of sulfur and potassium *tert*-butoxide, but only one example was shown [35]. In 2017, the Lei group reported a copper catalyzed C–H mercaptalization strategy using elementary sulfur as thiol source [36]. This conversion proceeds under very mild conditions, however, a metal catalyst and an additional ligand are required (Scheme 1).

Although some protocols have been developed, these methods still suffer from some drawbacks, such as limited substrate scope, low yield, and/or a complicated reaction system. Accordingly, developing a new and simple method for the synthesis of 2-mercaptobenzothiazoles and 2-mercaptobenzoxazoles is still desirable. As a continuous study on C–S coupling reactions using aliphatic dithiols, herein we reported a simple and effective method for converting benzothiazoles and benzoxazoles to the corresponding thiols through direct C–H mercaptalization using 1,3-propanedithiol as thiol source.

## Results and Discussion

Previous studies in our group revealed that small aliphatic diols and dithiols are promising reagents for the synthesis of phenols and arylthiols, respectively [23,37]. Therefore, we envisioned that aliphatic dithiol may be able to work as thiol source in the C–H mercaptalization of benzothiazole and benzoxazole as well, leading to the formation of 2-mercaptobenzothiazole and 2-mercaptobenzoxazole, respectively. We tested our hypothesis using benzothiazole (**1a**) as model substrate together with several aliphatic dithiols **2**. Initially, benzothiazole was treated with 3.0 equiv of each aliphatic dithiol and 5.0 equiv of KOH in DMSO at 130 °C. After 12 h, we are delighted to find that **1a** was predominantly converted to 2-mercaptobenzothiazole (**3a**). The investigation of the reaction mixture by proton nuclear magnetic resonance spectroscopy showed that no byproduct was formed. It should be noted that the length of aliphatic dithiols had a significant effect on the reaction performance. The reaction with 1,3-propanedithiol (**2b**) showed the best reaction performance, providing **3a** with an isolated yield of 88% while the reaction with 1,2-ethanedithiol (**2a**) and 1,4-butanedithol (**2c**) gave yields of 36% and 45%, respectively (Table 1, entries 1–3). In the control reaction without aliphatic dithiol, **3a** was not observed (Table 1, entry 4).

**Table 1:** Screening of the conditions for C–H mercaptalization of benzothiazole.^a^

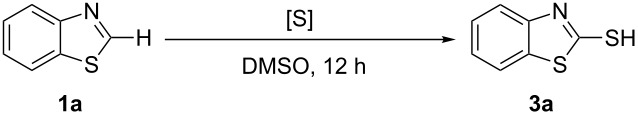				

Entry	Base (equiv)	Thiol surrogate (equiv)	Temp (°C)	Yield^b^ (%)

1	KOH (5)	**2a** (3)	130	36
2	KOH (5)	**2b** (3)	130	88, 79^c^
3	KOH (5)	**2c** (3)	130	45
4	KOH (5)	**–**	130	0
5	KOH (5)	**2b** (3)	120	20
6	KOH (5)	**2b** (3)	110	0
7	NaO*t*-Bu (5)	**2b** (3)	130	80
8	K_2_CO_3_(5)	**2b** (3)	130	8
9	Cs_2_CO_3_ (5)	**2b** (3)	130	67^d^
10	KOH (5)	**2b** (2)	130	92
11	KOH (5)	**2b** (1)	130	46
12	KOH (3)	**2b** (2)	130	78
13^e^	KOH (5)	**2b** (3)	130	8
14	KOH (5)	1-butanethiol (2)	130	15
15	KOH (5)	S (2)	130	21
16	KOH (5)	Na_2_S_2_O_3_ (2)	130	6
17	KOH (5)	Na_2_S·9H_2_O (2)	130	14
18	KOH (5)	K_2_S (2)	130	12
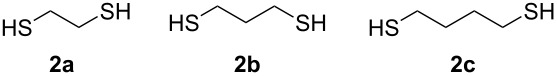				

^a^Reaction conditions: benzothiazole (**1a**, 1 mmol), thiol surrogate, base, DMSO (3 mL), 12 h. ^b^Isolated yield. ^c^6 h. ^d^5 mL of DMSO. ^e^DMF as solvent.

Shortening the reaction time to 6 h provided 79% yield of **3a** (Table 1, entry 2). Lowering of the reaction temperature led to lower yields. No product was observed at 110 °C while 20% yield of **3a** was obtained at 120 °C (Table 1, entries 5 and 6). The investigation of different bases revealed that KOH was the most effective in this reaction in comparison to other bases such as NaO*t*-Bu, K_2_CO_3_ and Cs_2_CO_3_ (Table 1, entries 7–9). Using 2.0 equiv of 1,3-propanedithiol did not lead to the loss of yield, however, only 46% yield was obtained when the amount of 1,3-propanedithiol was further lowered to 1.0 equiv. Five equivalents of KOH were required for this transformation as only 78% yield was obtained when 3.0 equiv of KOH was used (Table 1, entries 10–12). DMSO was essential for this reaction because replacing DMSO with another organic solvent such as DMF significantly decreased the reaction yield to 8% (Table 1, entry 13). Therefore, the optimized reaction conditions were obtained as follows: benzothiazole (1.0 mmol), 1,3-propanedithiol (2.0 equiv), KOH (5.0 equiv), DMSO (3 mL), 130 °C, 12 h.

We further investigated several common thiol surrogates, which are often used in the C–H mercaptalization of aryl halides. Under the optimized conditions, 1-butanethiol gave only 15% yield of **3a** and many byproducts were formed (Table 1, entry 14). Other thiol surrogates including elementary sulfur, Na_2_S_2_O_3,_ Na_2_S·9H_2_O and K_2_S also provided very low yields (Table 1, entries 15–18). These results show that 1,3-propanedithiol is a promising thiol source for C–H mercaptalization of benzothiazole.

**Figure 2:**
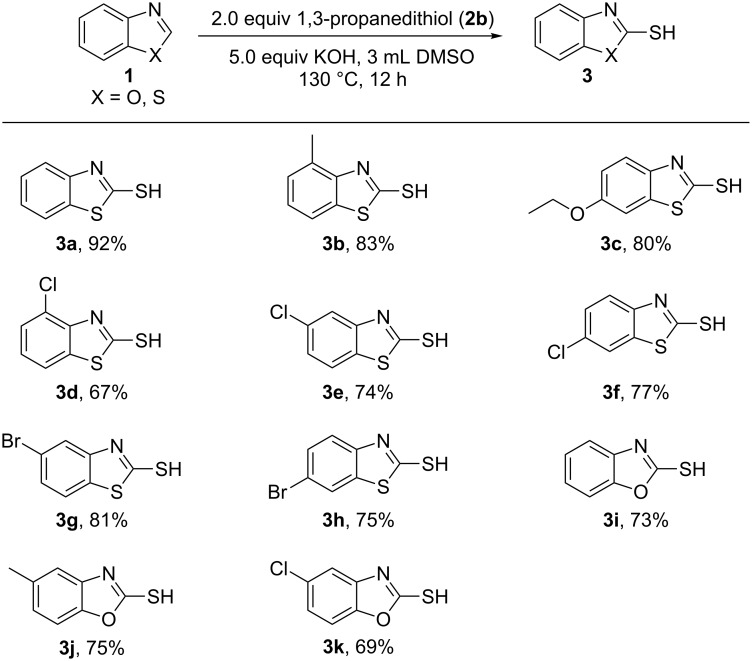
Substrate scope of the developed C–H mercaptalization strategy. Reaction conditions: benzothiazole or benzoxazole **1** (1.0 mmol), 1,3-propanedithiol (**2b**, 2.0 equiv), KOH (5.0 equiv), DMSO (3 mL), 130 °C, 12 h.

With the optimized conditions in hand, we studied the substrate scope for this novel C–H mercaptalization strategy (Figure 2). Generally, benzothiazoles were converted to the corresponding heteroarylthiols in moderate to good yields. Functional groups including methyl and ethoxy groups as well as halogens are well tolerated under the developed reaction conditions. Benzoxazoles were also successfully converted to the corresponding thiols. The relatively lower yields can be attributed to the partial decomposition of benzoxazoles caused by KOH at high temperature.

**Scheme 2:**
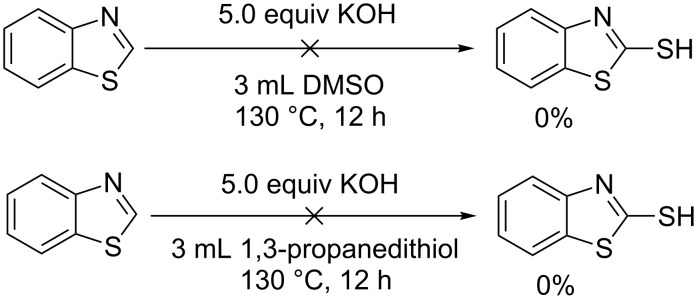
Control experiments.

In order to get more understanding of this novel C–H mercaptalization strategy, several control experiments were carried out (Scheme 2). 2-Mercaptobenzothiazole was not observed when the reaction was carried out in the absence of either 1,3-propanedithiol or DMSO. This result indicate that 1,3-propanedithiol may react with DMSO and give an active intermediate, which can further convert benzothiazole to 2-mercaptobenzothiazole. Indeed, several articles have reported that thiols can be oxidized by DMSO to the corresponding disulfides [38–39].

**Scheme 3:**
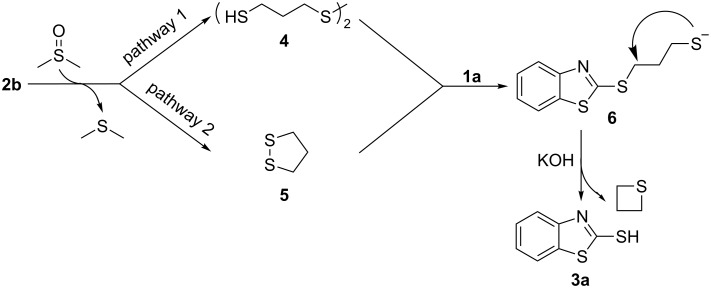
Proposed reaction pathway.

Based on the above results and related references, a plausible reaction pathway is proposed (Scheme 3). Initially, 1,3-propanedithiol is possibly oxidized to disulfides **4** and **5** by DMSO. We failed to isolate and determinate these two sulfides, possibly because they are very active in the following coupling reactions. Both disulfides coupled with **1a** to give the same C–S coupling product **6** [40]. As our previous work shows [23], (hetero)arylthioalkylthiols are easily converted to the corresponding (hetero)arylthiols in the presence of KOH and DMSO through an intramolecular nucleophilic substitution.

## Conclusion

In this work, we developed a facile protocol for the direct synthesis of 2-mercaptobenzothiazoles and 2-mercaptobenzoxazoles from benzothiazoles and benzoxazoles. 1,3-Propanedithiol served as a thiol source and was superior to other common thiol surrogates under our developed conditions. DMSO was indispensable for this conversion and a preliminary mechanism study showed it served not only as a solvent but also as an oxidant. The developed reaction system required neither a metal catalyst nor a ligand. This simple method is expected to have potential application in both laboratory and industry.

## Supporting Information

File 1 General experimental information, synthetic procedures, analytical data and NMR spectra for the reported compounds.

## References

[1] Gill R K, Rawal R K, Bariwal J (2015). Arch Pharm (Weinheim, Ger).

[2] Ranjit S, Lee R, Heryadi D, Shen C, Wu J, Zhang P, Huang K-W, Liu X (2011). J Org Chem.

[3] Yarar S, Ozturk I I, Banti C N, Panagiotou N, Papatriantafyllopoulou C, Manoli M, Manos M J, Tasiopoulos A J, Hadjikakou S K (2018). Inorg Chim Acta.

[4] El-Asmy H A, Butler I S, Mouhri Z S, Jean-Claude B J, Emmam M, Mostafa S I (2016). Inorg Chim Acta.

[5] Mitra R, Samuelson A G (2014). Eur J Inorg Chem.

[6] Prasad A V G S, Rao P V, Prasad P S S (2014). Int J Pharm Res Scholars.

[7] Satyendra R V, Vishnumurthy K A, Vagdevi H M, Dhananjaya B L, Shruthi A (2015). Med Chem Res.

[8] Defrenza I, Catalano A, Carocci A, Carrieri A, Muraglia M, Rosato A, Corbo F, Franchini C (2015). J Heterocycl Chem.

[9] Hornung M W, Kosian P A, Haselman J T, Korte J J, Challis K, Macherla C, Nevalainen E, Degitz S J (2015). Toxicol Sci.

[10] Testaferri L, Tingoli M, Tiecco M (1980). Tetrahedron Lett.

[11] Testaferri L, Tiecco M, Tingoli M, Chianelli D, Montanucci M (1983). Synthesis.

[12] Shaw J E (1991). J Org Chem.

[13] Liu Y, Liu S, Xiao Y (2017). Beilstein J Org Chem.

[14] Wang F, Cai S, Wang Z, Xi C (2011). Org Lett.

[15] Varun B V, Prabhu K R (2014). J Org Chem.

[16] Lou C, Zhu N, Fan R, Hong H, Han L, Zhang J, Suo Q (2017). Green Chem.

[17] Deligeorgiev T G, Kaloyanova S S, Lesev N Y, Vaquero J J (2011). Monatsh Chem.

[18] Liu L, Zhu N, Gao M, Zhao X, Han L, Hong H (2016). Phosphorus, Sulfur Silicon Relat Elem.

[19] Liu X, Liu M, Xu W, Zeng M-T, Zhu H, Chang C-Z, Dong Z-B (2017). Green Chem.

[20] Zhu X, Li W, Luo X, Deng G, Liang Y, Liu J (2018). Green Chem.

[21] Foye W O, Abood N, Kauffman J M, Kim Y-H, Patel B R (1980). Phosphorus Sulfur Relat Elem.

[22] Watt G W (1939). J Org Chem.

[23] Liu Y, Kim J, Seo H, Park S, Chae J (2015). Adv Synth Catal.

[24] Shen C, Zhang P, Sun Q, Bai S, Hor T S A, Liu X (2015). Chem Soc Rev.

[25] Lee C-F, Basha R S, Badsara S S (2018). Top Curr Chem.

[26] Dai C, Xu Z, Huang F, Yu Z, Gao Y-F (2012). J Org Chem.

[27] Rosario A R, Casola K K, Oliveira C E S, Zeni G (2013). Adv Synth Catal.

[28] Rafique J, Saba S, Frizon T E A, Braga A L (2018). ChemistrySelect.

[29] He Z, Luo F, Li Y, Zhu G (2013). Tetrahedron Lett.

[30] Gandeepan P, Mo J, Ackermann L (2017). Chem Commun.

[31] Liu Y, Wang H, Wang C, Wan J-P, Wen C (2013). RSC Adv.

[32] Zhou A-X, Liu X-Y, Yang K, Zhao S-C, Liang Y-M (2011). Org Biomol Chem.

[33] Inomata H, Toh A, Mitsui T, Fukuzawa S-i (2013). Tetrahedron Lett.

[34] Fukuzawa S-i, Shimizu E, Atsuumi Y, Haga M, Ogata K (2009). Tetrahedron Lett.

[35] Popov I, Do H-Q, Daugulis O (2009). J Org Chem.

[36] Yan H, Huang Z, Chen M, Li C, Chen Y, Gao M, Lei A (2017). Org Biomol Chem.

[37] Liu Y, Park S K, Xiao Y, Chae J (2014). Org Biomol Chem.

[38] Le Quéméner F, Subervie D, Morlet-Savary F, Lalevée J, Lansalot M, Bourgeat-Lami E, Lacôte E (2018). Angew Chem, Int Ed.

[39] Liu X G, Wu J P, Liang X M, Wang D Q (2001). Chin J Org Chem.

[40] Zou L-H, Reball J, Mottweiler J, Bolm C (2012). Chem Commun.

