# Supplementary material for: Metal-free C–H mercaptalization of benzothiazoles and benzoxazoles using 1,3-propanedithiol as thiol source
Source: Beilstein J Org Chem. 2019 Jan 29;15:279–84. doi: 10.3762/bjoc.15.24 (PMC6369980; doi:10.3762/bjoc.15.24)
Supplement: File 1 — General experimental information, synthetic procedures, analytical data and NMR spectra for the reported compounds. [file Beilstein_J_Org_Chem-15-279-s001.pdf]

BEILSTEIN JOURNAL OF ORGANIC CHEMISTRY

Supporting Information

for

Metal-free C–H mercaptalization of benzothiazoles and benzoxazoles using 1,3-propanedithiol as thiol source

Yan Xiao, Bing Jing, Xiaoxia Liu, Hongyu Xue and Yajun Liu

Beilstein J. Org. Chem. **2019,** *15,* 279–284. doi:10.3762/bjoc.15.24

General experimental information, synthetic procedures, analytical data and NMR spectra for the reported compounds

License and Terms: This is a supporting information file under the terms of the Creative Commons Attribution License (http://creativecommons.org/licenses/by/4.0). Please note that the reuse, redistribution and reproduction in particular requires that the authors and source are credited.
The license is subject to the *Beilstein Journal of Organic Chemistry* terms and conditions: (https://www.beilstein-journals.org/bjoc)

1. Experimental section

General information: All chemicals were purchased as reagent grade and used without further purification. Solvents for purification (extraction and chromatography) were purchased as technical grade and distilled on the rotary evaporator prior to use. For column chromatography, SiO_2 (200–300 mesh) was used as stationary phase. NMR spectra were recorded on a 500 MHz Bruker NMR spectrometer with tetramethylsilane (TMS) as an internal standard.

General procedure for C–H mercaptalization of benzothiazoles and benzoxazoles: To a solution of benzothiazoles or benzoxazoles (**1**, 1 mmol) in DMSO (3 mL), was added KOH (280 mg, 5 equiv) and 1,3-propanedithiol (207 μL, 2 mmol). The reaction mixture was heated under argon at 130 °C for 12 h. After cooling to room temperature, water (10 mL) was added. The pH of the reaction mixture was adjusted to 3–4 using 5% HCl. The resulting mixture was extracted with ethyl acetate (15 mL × 2). The organic layer was washed with water and brine, dried over anhydrous $MgSO_4$ and concentrated using rotary evaporator. The crude product was purified by silica gel column chromatography using ethyl acetate/*n*-hexane as eluent to afford the corresponding heteroaryl thiols **3**.

Benzo[*d*]thiazole-2-thiol [1] (**3a**) [CAS Registry Number: 149-30-4], yellow solid, 153 mg, yield 92%. ^{1}H NMR (500 MHz, DMSO-d_6) 13.77 (s, 1H), 7.70 (d, *J*= 8.0 Hz, 1H), 7.40 (t, *J*= 8.0 Hz, 1H), 7.32-7.27 (m, 2H); ^{13}C NMR (126 MHz, DMSO-d_6) 190.3, 141.7, 129.8, 127.6, 124.7, 122.3, 112.9.

4-Methylbenzo[*d*]thiazole-2-thiol[2] [CAS Registry Number: 2268-77-1] (**3b**), yellow solid, 150 mg, yield 83%. ^{1}H NMR (500 MHz, $CDCl_3$) 10.94 (s, 1H), 7.30 (d, *J*= 7.5 Hz, 1H), 7.21-7.15 (m, 2H), 2.49 (s, 3H); ^{13}C NMR (126 MHz, $CDCl_3$) 190.8, 139.3, 129.7, 128.3, 124.7, 122.0, 118.9, 17.9.

6-Ethoxybenzo[*d*]thiazole-2-thio l[1] (**3c**) [CAS Registry Number: 120-53-6], yellow solid, 169 mg, yield 80%. ^{1}H NMR (500 MHz, DMSO-d_6) 13.62 (s, 1H), 7.33 (d, *J*= 6.0 Hz, 1H), 7.22 (d, *J*= 8.5 Hz, 1H), 6.98 (dd, *J*= 2.0, 8.5 Hz, 1H), 4.02 (q, *J*= 7.0 Hz, 2H), 1.33 (t, *J*= 7.0 Hz, 3H) ; ^{13}C NMR (126 MHz, DMSO-d_6) 188.8, 156.3, 135.6, 131.1, 115.7, 113.6, 107.0, 64.2, 15.1.

4-Chlorobenzo[d]thiazole-2-thiol [3] (**3d**) [CAS Registry Number: 1849-65-6], orange solid, 135 mg, yield 67%. ^{1}H NMR (500 MHz, DMSO-d_6) 13.97 (s, 1H), 7.67 (d, *J*= 8.0 Hz, 1H), 7.48 (d, *J*= 7.5 Hz, 1H),7.30 (t, *J*= 8.0 Hz, 1H); ^{13}C NMR (126 MHz, DMSO-d_6) 191.6, 139.2, 127.7, 125.6, 121.0, 116.3.

5-Chlorobenzo[d]thiazole-2-thiol [3] (**3e**) [CAS Registry Number: 5331-91-9], orange solid, 149 mg, yield 74%. ^{1}H NMR (500 MHz, $CDCl_3$) 10.42 (s, 1H), 7.37-7.35 (m, 1H), 7.29-7.24 (m, 1H), 7.21-7.18 (m, 1H); ^{13}C NMR (126 MHz, $CDCl_3$) 180.9, 148.8, 130.2, 125.3, 124.4, 110.6, 110.0.

6-Chlorobenzo[d]thiazole-2-thiol [3] (**3f**) [CAS Registry Number: 51618-29-2], orange solid, 155 mg, yield 77%. ^{1}H NMR (500 MHz, DMSO-d_6) 13.88 (s, 1H), 7.87 (d, *J*= 1.5 Hz, 1H), 7.45 (dd, *J*= 2.0, 8.5 Hz, 1H), 7.29 (d, *J*= 8.5 Hz, 1H);^{13}C NMR (126 MHz, DMSO-d_6) 190.6, 140.7, 131.5, 129.1, 127.8, 121.9, 114.0.

5-Bromobenzo[d]thiazole-2-thiol [4] (**3g**) [CAS Registry Number: 71216-20-1], orange solid, 200 mg, yield 81%. ^{1}H NMR (500 MHz, DMSO-d_6) 13.97 (s, 1H), 7.67 (d, *J*= 8.0 Hz, 1H), 7.48 (d, *J*= 8.0 Hz, 1H), 7.32-7.28 (m, 1H); ^{13}C NMR (126 MHz, DMSO-d_6) 191.6, 139.2, 131.2, 127.7, 125.6, 121.0, 116.3.

6-Bromobenzo[d]thiazole-2-thiol [3] (**3h**) [CAS Registry Number: 51618-30-5], orange solid, 185 mg, yield 75%. ^{1}H NMR (500 MHz, DMSO-d_6) 13.88 (s, 1H), 7.99 (d, *J*= 1.5 Hz, 1H), 7.57 (dd, *J*= 1.5, 8.5 Hz, 1H), 7.23 (d, *J*= 8.5 Hz, 1H); ^{13}C NMR (126 MHz, DMSO-d_6) 190.6, 141.1, 131.9, 130.5, 124.7, 116.8, 114.4.

Benzo[d]oxazole-2-thiol [5] (**3i**) [CAS Registry Number: 2382-96-9], yellow solid, 110 mg, yield 73%. ^{1}H NMR (500 MHz, DMSO-d_6) 13.87 (s, 1H), 7.51 (d, *J*= 7.5 Hz, 1H), 7.30-7.23 (m, 3H); ^{13}C NMR (126 MHz, DMSO-d_6) 180.6, 148.6, 131.6, 125.6, 124.3, 111.0, 110.5.

5-Methylbenzo[d]oxazole-2-thiol [5] (**3j**) [CAS Registry Number: 22876-22-8], yellow solid, 165 mg, yield 75%. ^{1}H NMR (500 MHz, $CDCl_3$) 10.36 (s, 1H), 7.22 (d, *J*= 8.0 Hz, 1H), 7.05 (d, J= 8.5 Hz, 1H), 7.00 (s, 1H), 2.42 (s, 3H); ^{13}C NMR (126 MHz, $CDCl_3$) 180.9, 147.0, 135.6, 130.2, 125.1, 110.2, 110.0, 21.4.

5-Chlorobenzo[d]oxazole-2-thiol [6] (**3k**) [CAS Registry Number: 22876-19-3], orange solid, 127 mg, yield 69%. ^{1}H NMR (500 MHz, DMSO-d_6) 14.05 (s, 1H), 7.53 (dd, *J*= 1.5, 7.5 Hz, 1H), 7.32-7.30 (s, 2H); ^{13}C NMR (126 MHz, DMSO-d_6) 181.2, 147.5, 133.1, 129.8, 124.0, 111.7, 110.9.

References:

[1] Cressier, D.; Prouillac, C.; Hernandez, P.; Amourette, C.; Diserbo, M.; Lion, C.; Rima, G. *Bioorg. Med. Chem.*, **2009**, *17*, 5275-5284.

[2] Murthi, Y.; Pathak, D. *J. Pharm. Res.*, **2008**, 7, 153-155.

[3] Huang, W.; Tan, Y.; Ding, M.-W.; Yang, G.-F. *Synth. Commun.*, **2007**, *37*, 369-376.

[4] Anima, B.; Hosahalli, S.; Subhendu, M. WO 2013042137.

[5] Venkatachalam, K. T.; Pierens, G. K.; Reutens, D. C. *Lett. Org. Chem.*, **2010**, *7*, 519-527.

[6] Baxter, C. A.; Cleator, E.; Brands, K. M. J.; Edwards, J. S.; Reamer, R. A.; Sheen, F. J.; Stewart, G. W.; Strotman, N. A.; Wallace, D. J. *Org. Process Res. Dev.*, **2011**, *15*, 367-375.

2. NMR spectra of compound 3a–k.

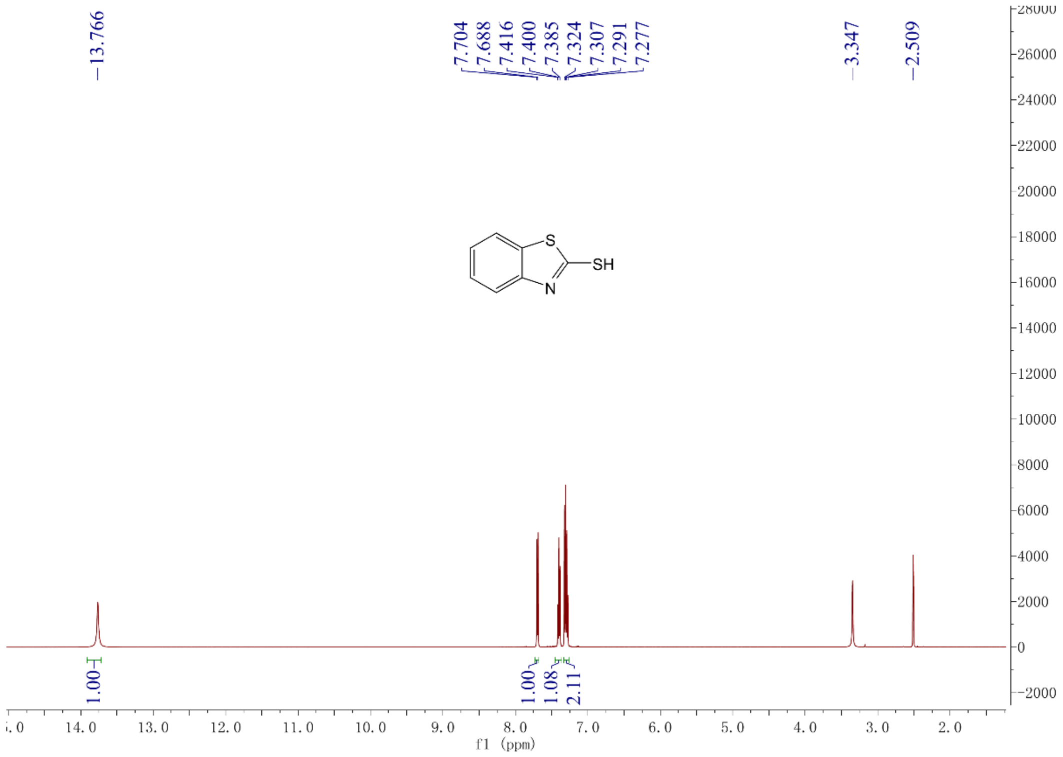

Figure 1: ^{1}H NMR spectrum of compound **3a**

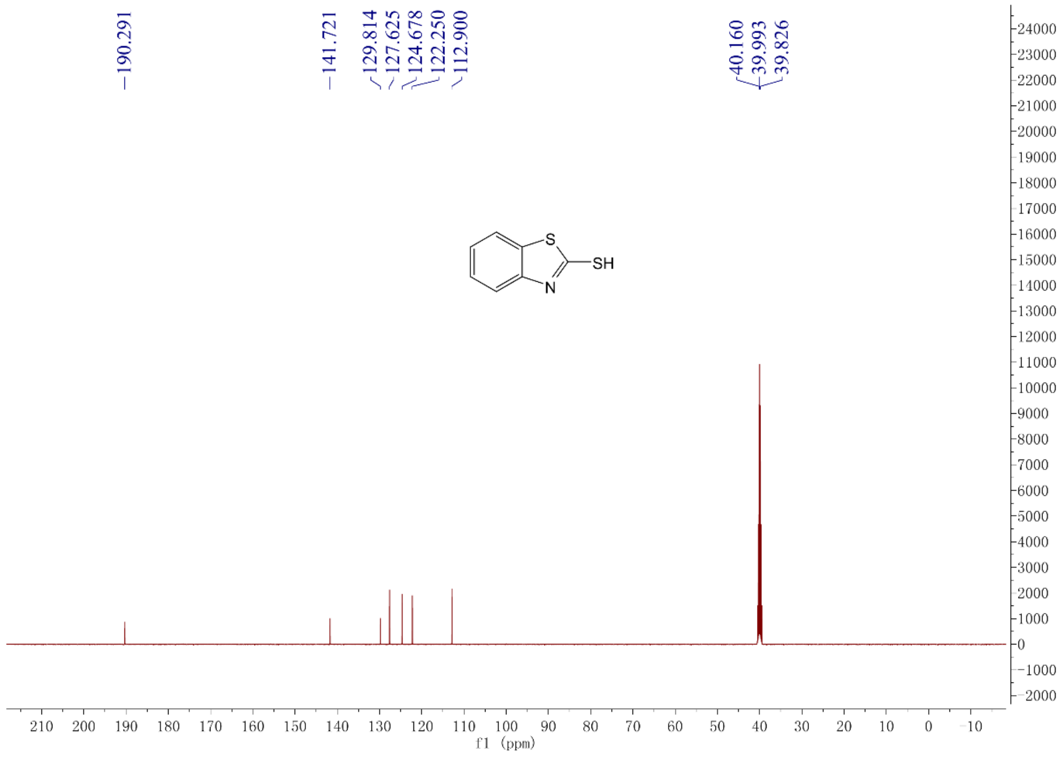

Figure 2: ^{13}C NMR spectrum of compound **3a**

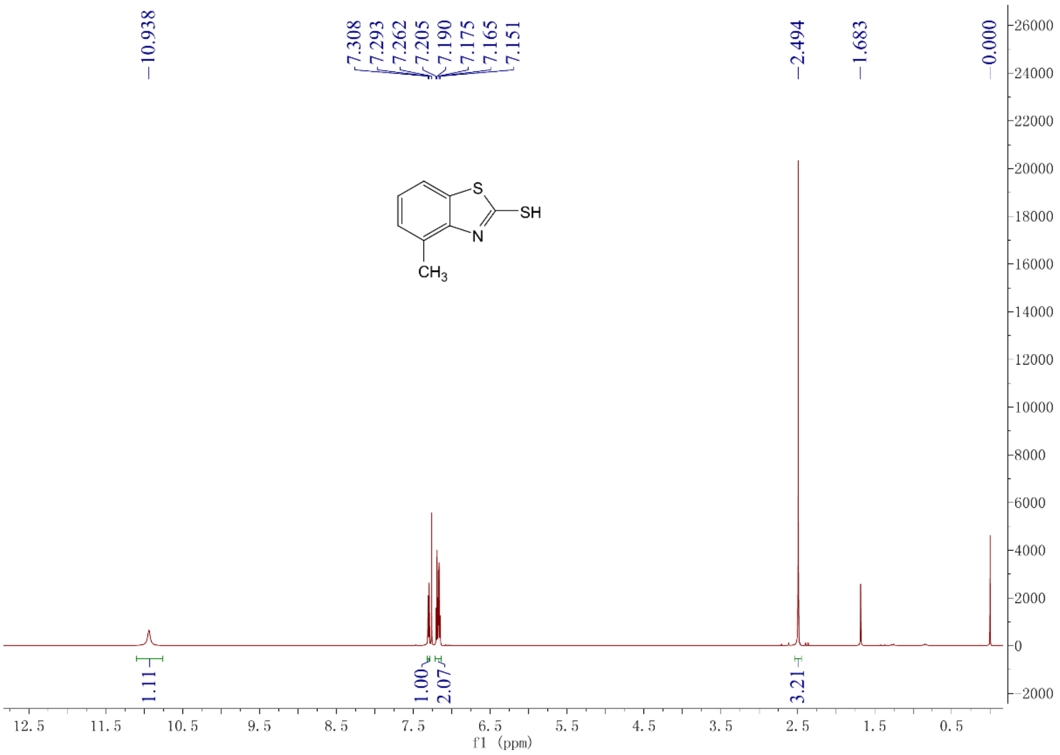

Figure 3: ^{1}H NMR spectrum of compound **3b**

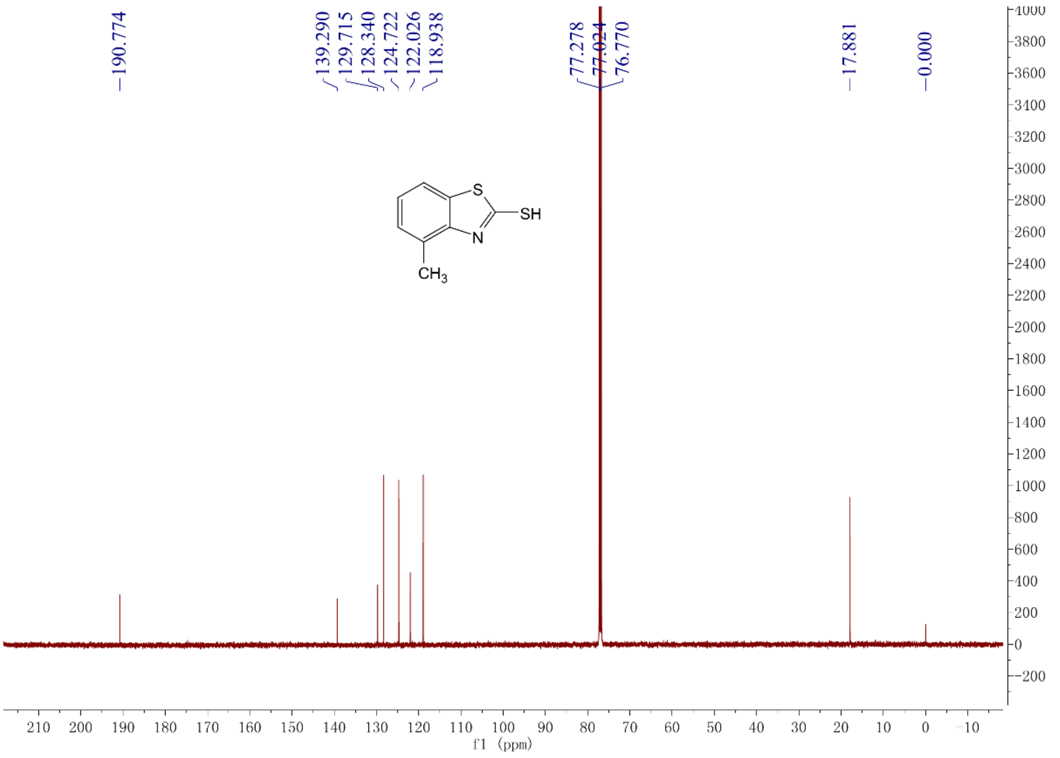

Figure 4: ^{13}C NMR spectrum of compound **3b**

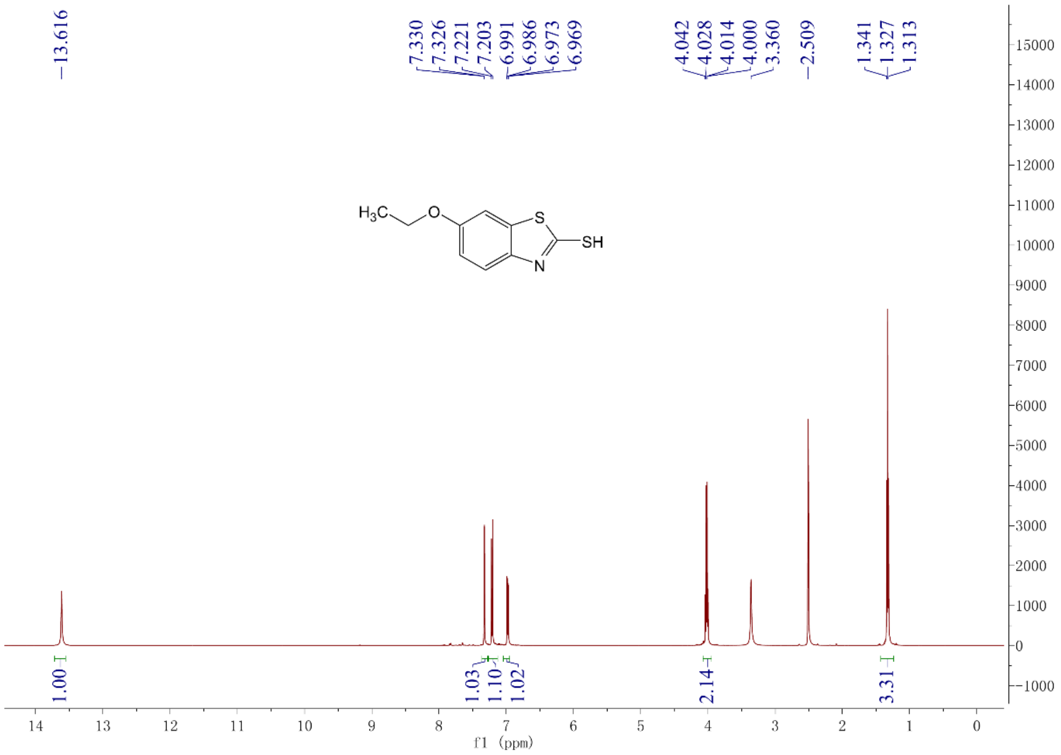

Figure 5: ^{1}H NMR spectrum of compound **3c**

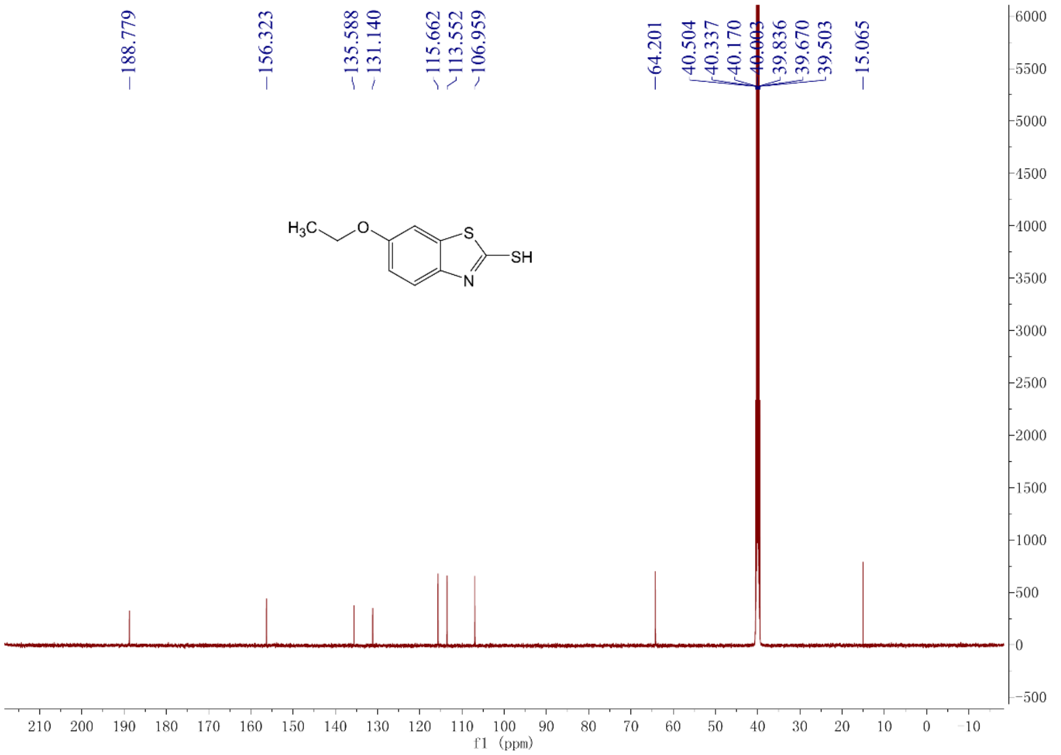

Figure 6: ^{13}C NMR spectrum of compound **3c**

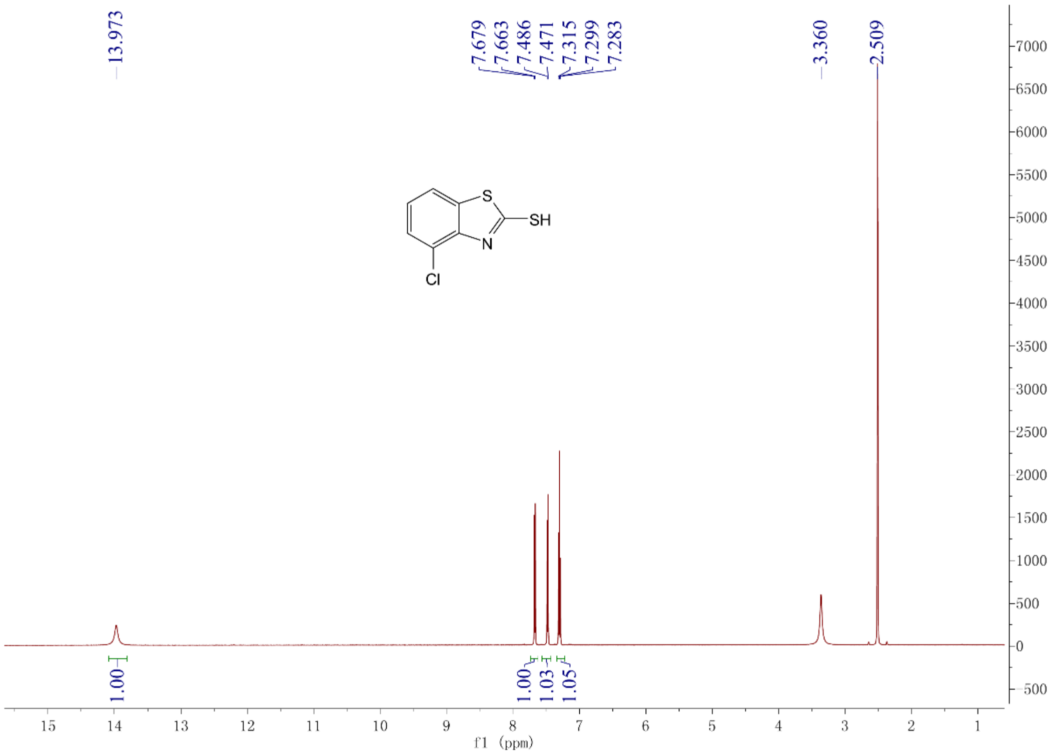

Figure 7: ^{1}H NMR spectrum of compound **3d**

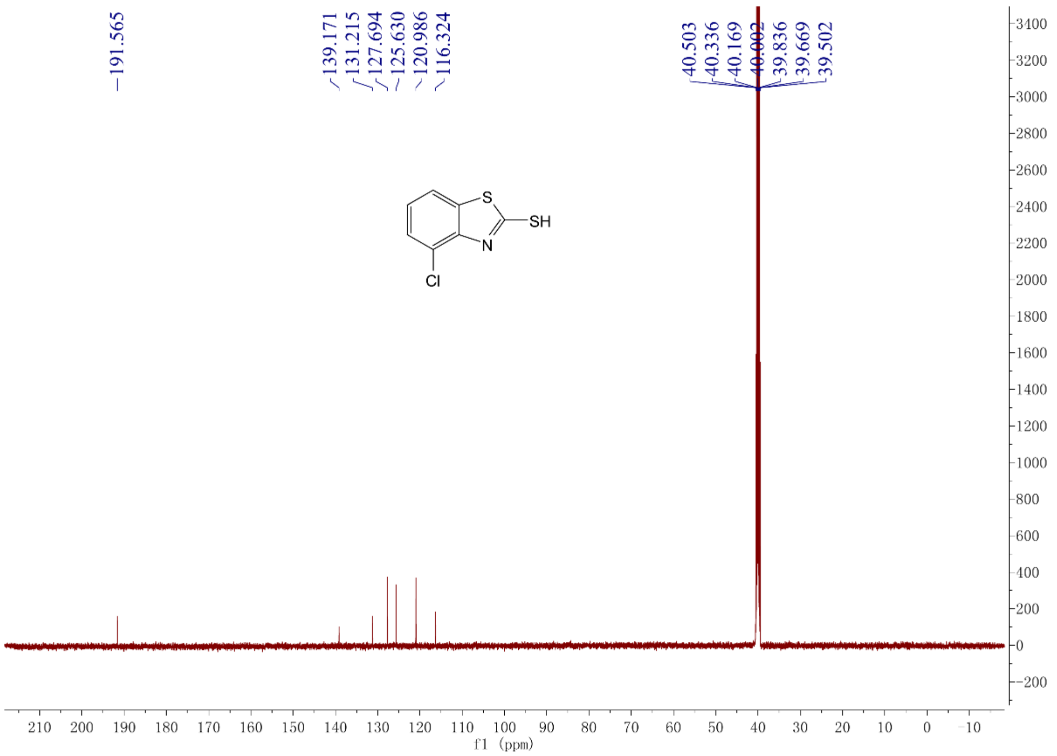

Figure 8: ^{13}C NMR spectrum of compound **3d**

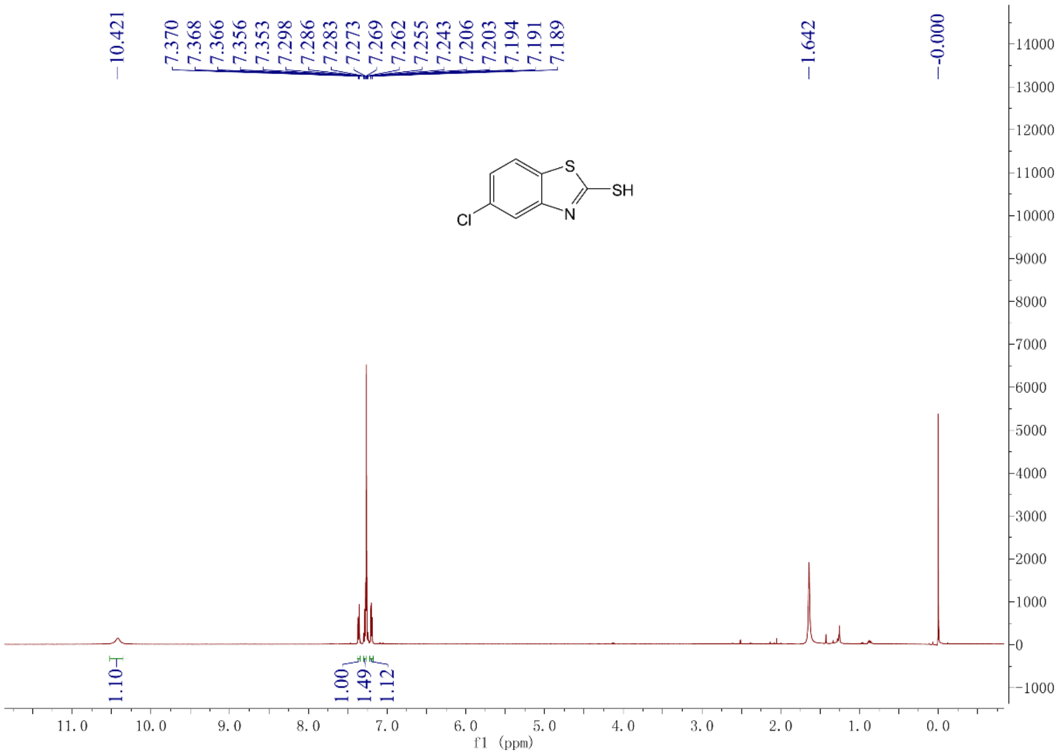

Figure 9: ^{1}H NMR spectrum of compound **3e**

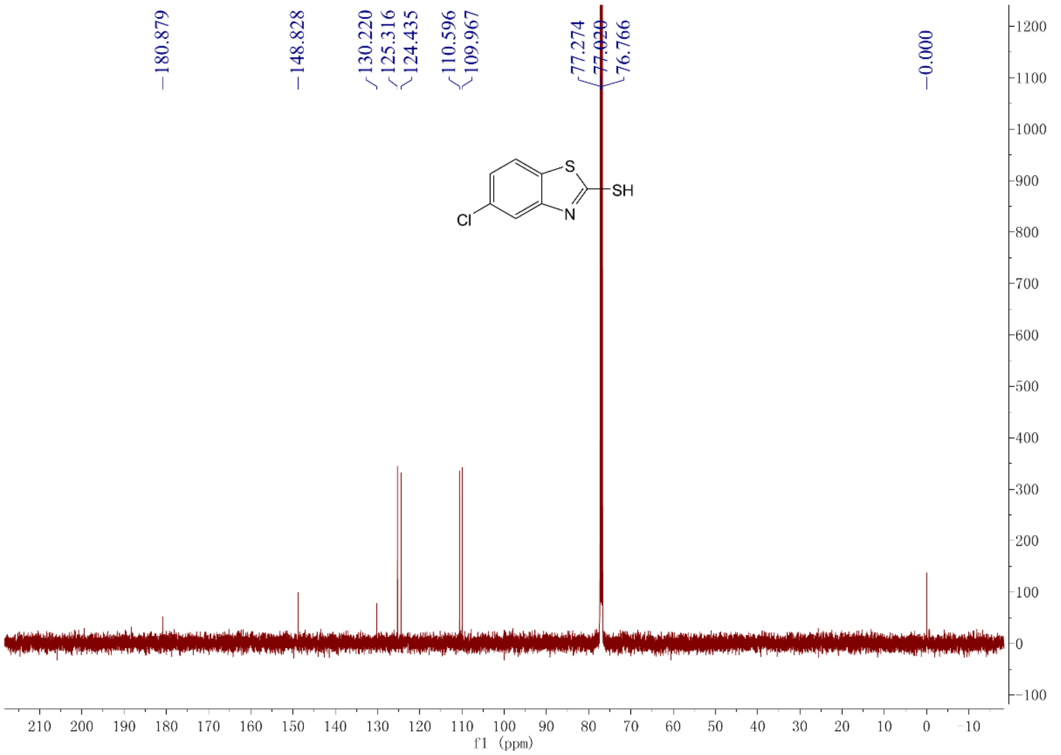

Figure 10: ^{13}C NMR spectrum of compound **3e**

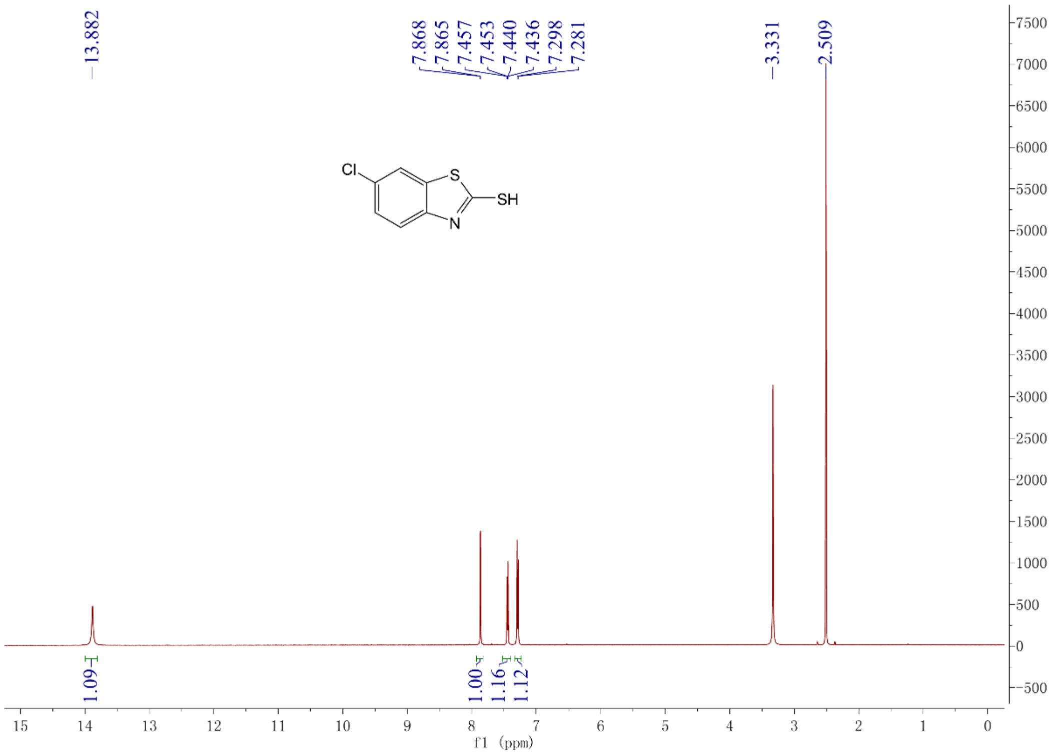

Figure 11: ^{1}H NMR spectrum of compound **3f**

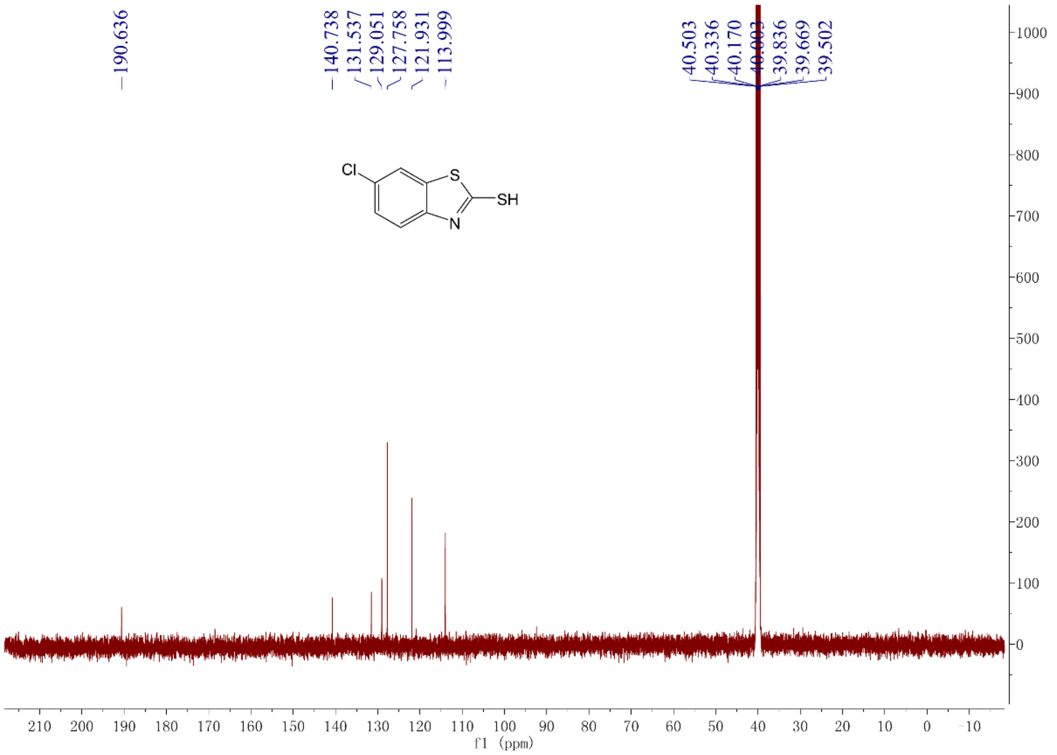

Figure 12: ^{13}C NMR spectrum of compound **3f**

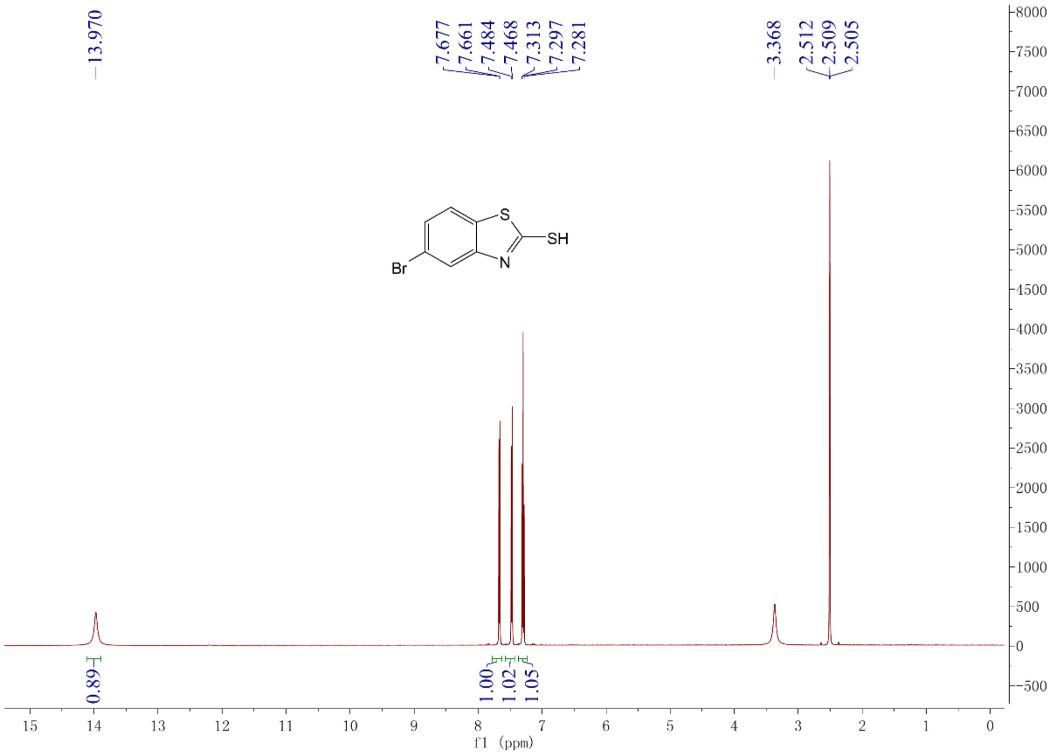

Figure 13: ^{1}H NMR spectrum of compound **3g**

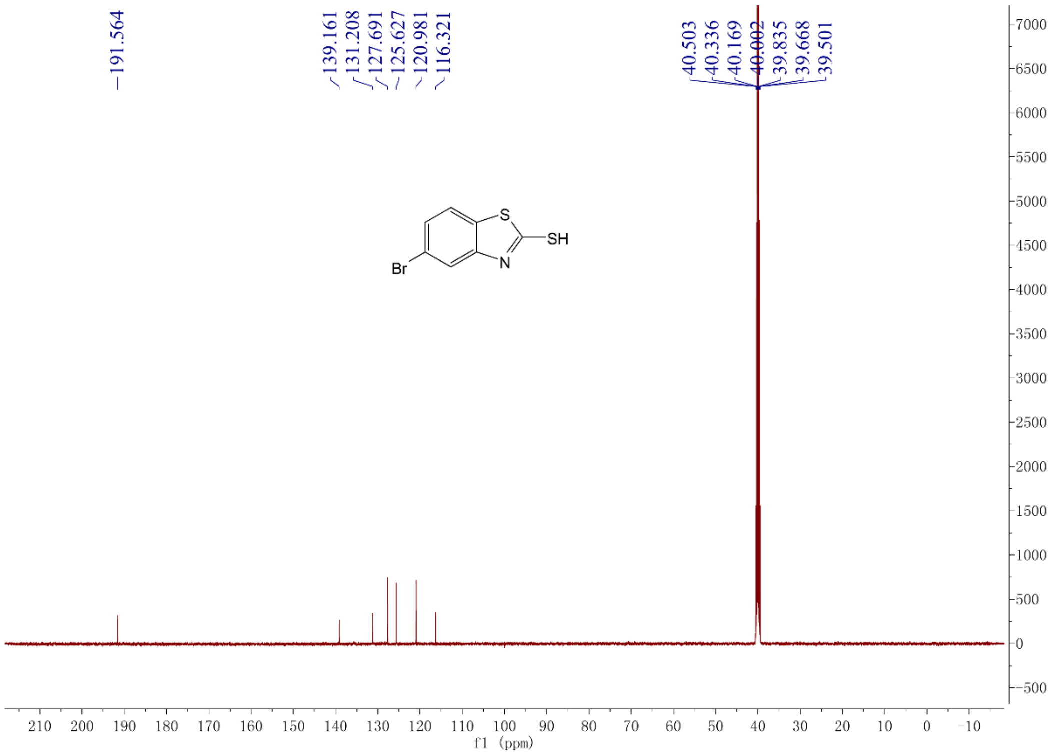

Figure 14: ^{13}C NMR spectrum of compound **3g**

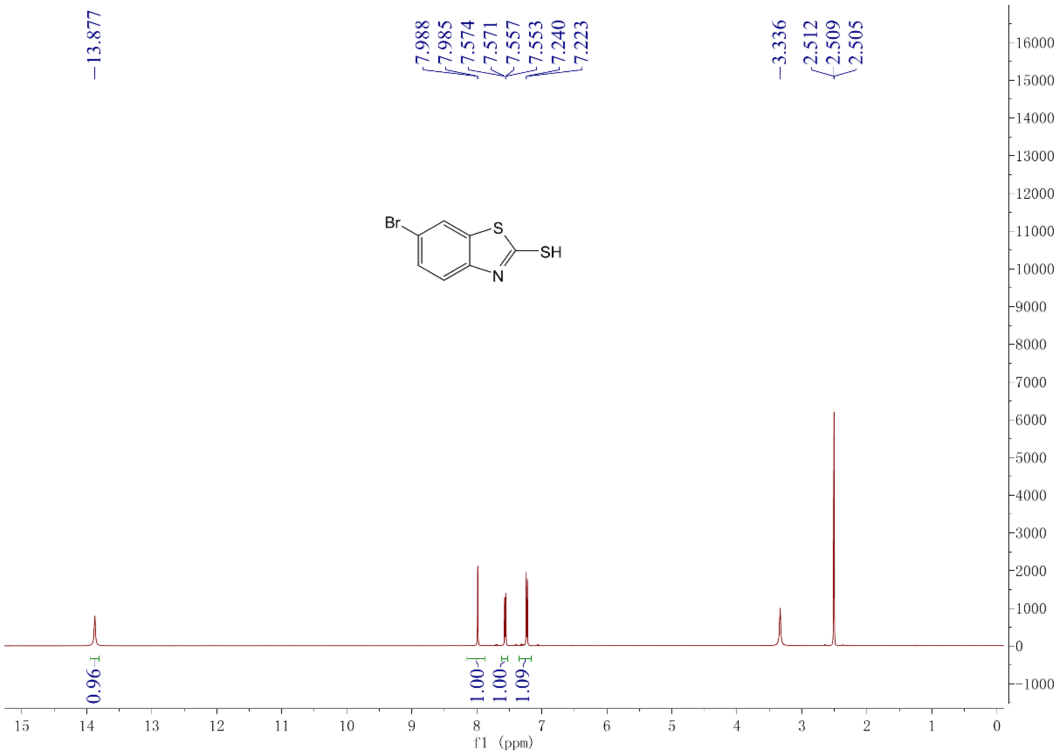

Figure 15: ^{1}H NMR spectrum of compound **3h**

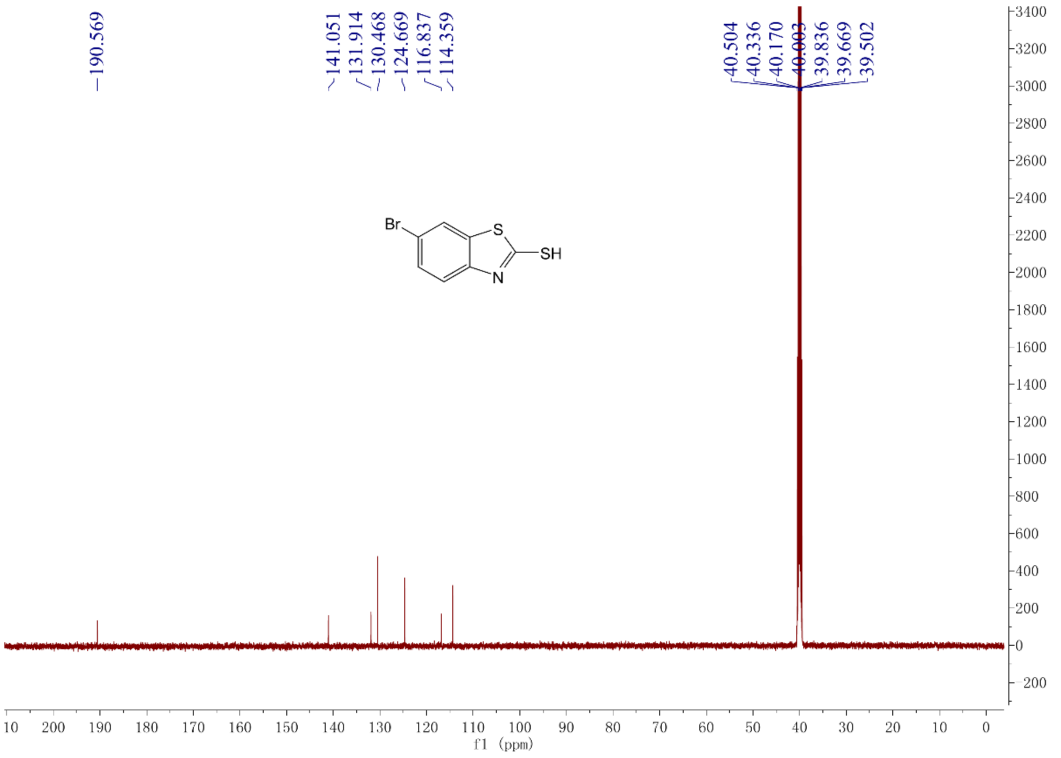

Figure 16: ^{13}C NMR spectrum of compound **3h**

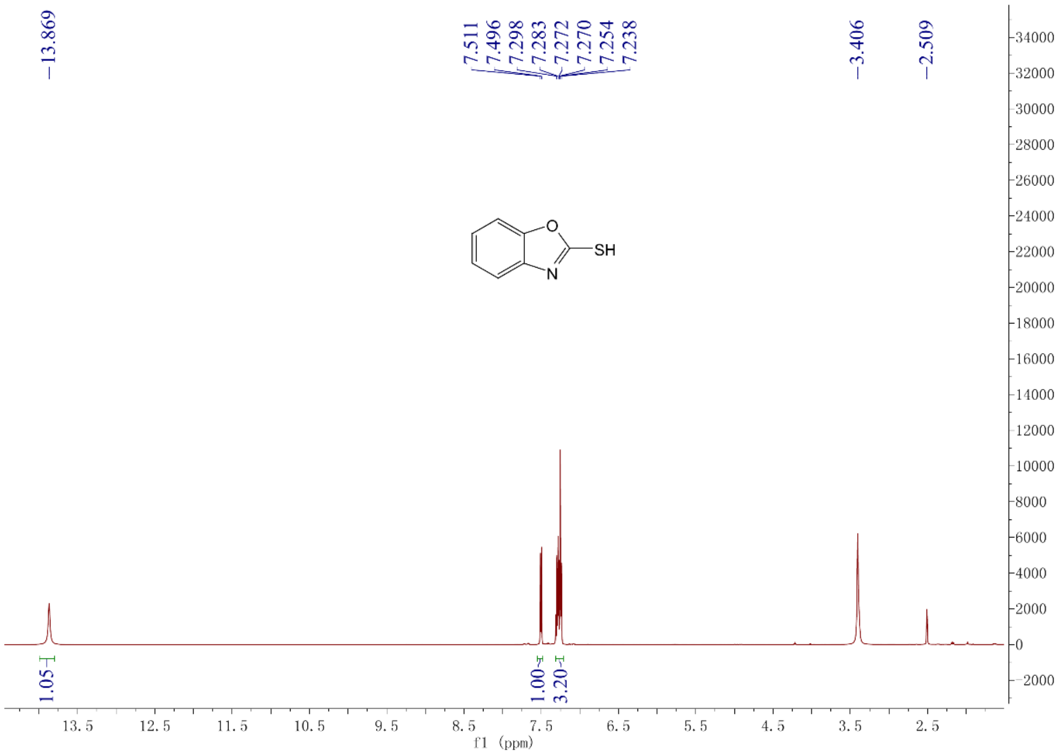

Figure 17: ^{1}H NMR spectrum of compound **3i**

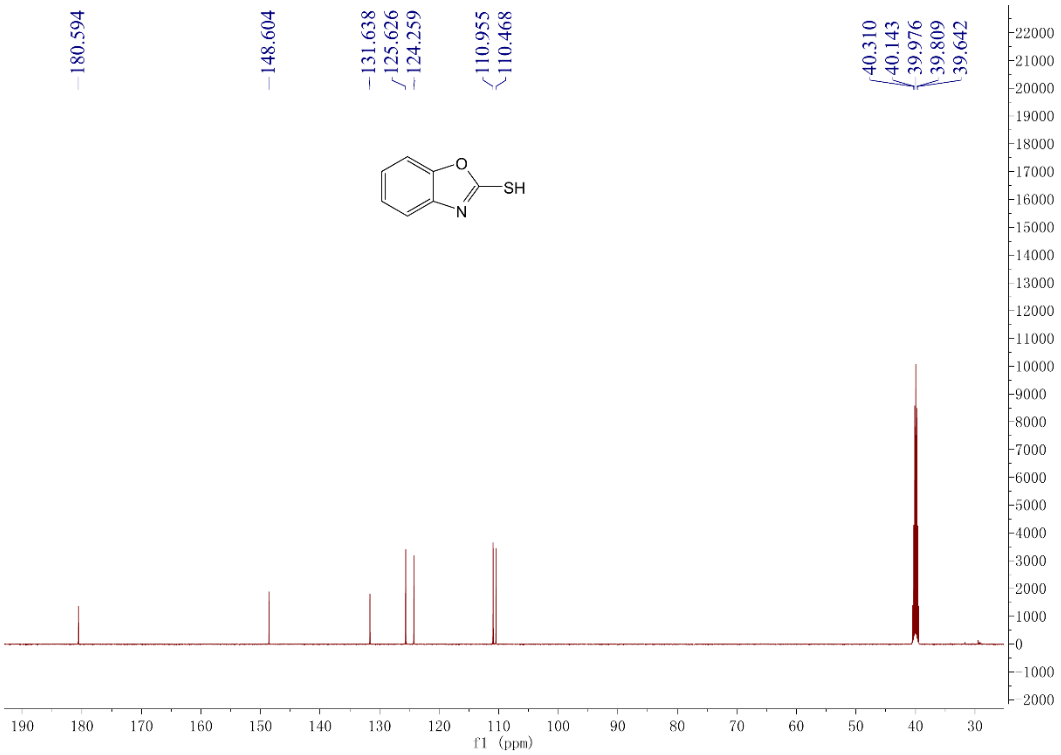

Figure 18: ^{13}C NMR spectrum of compound **3i**

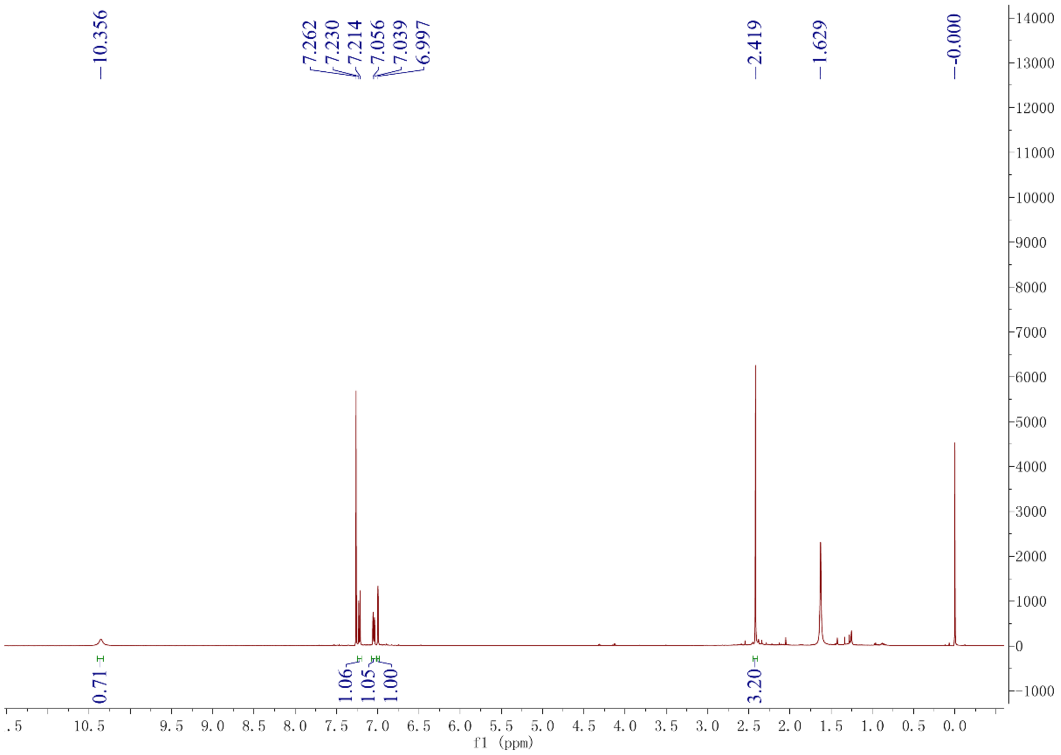

Figure 19: ^{1}H NMR spectrum of compound **3j**

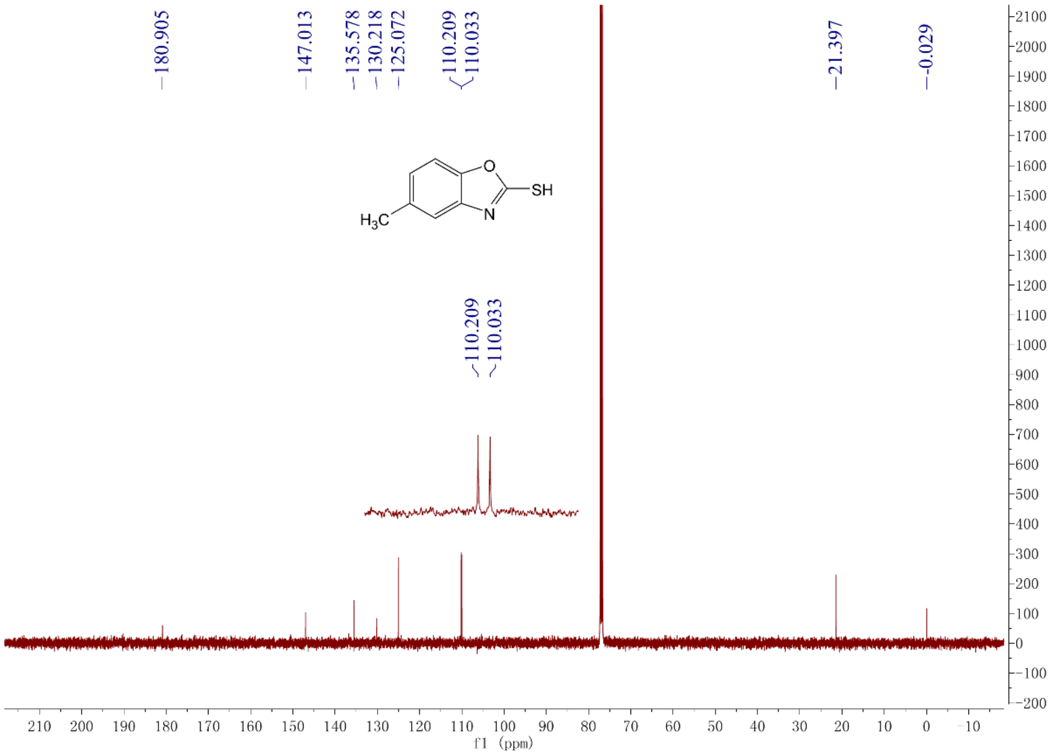

Figure 20: ^{13}C NMR spectrum of compound **3j**

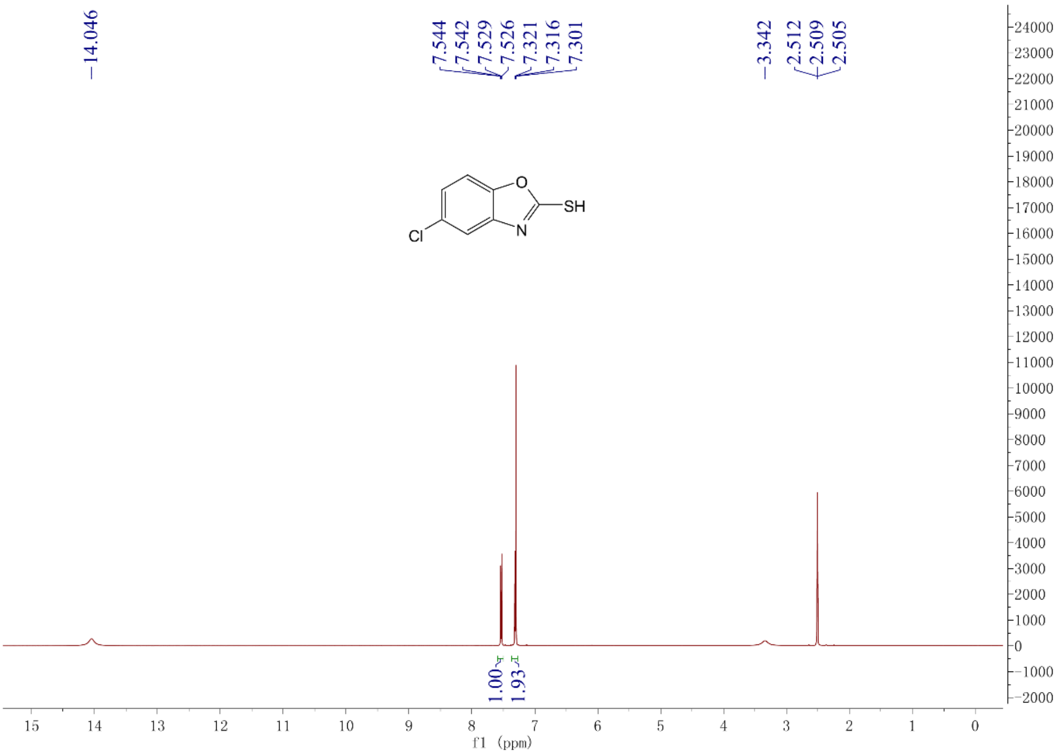

Figure 21: ^{1}H NMR spectrum of compound **3k**

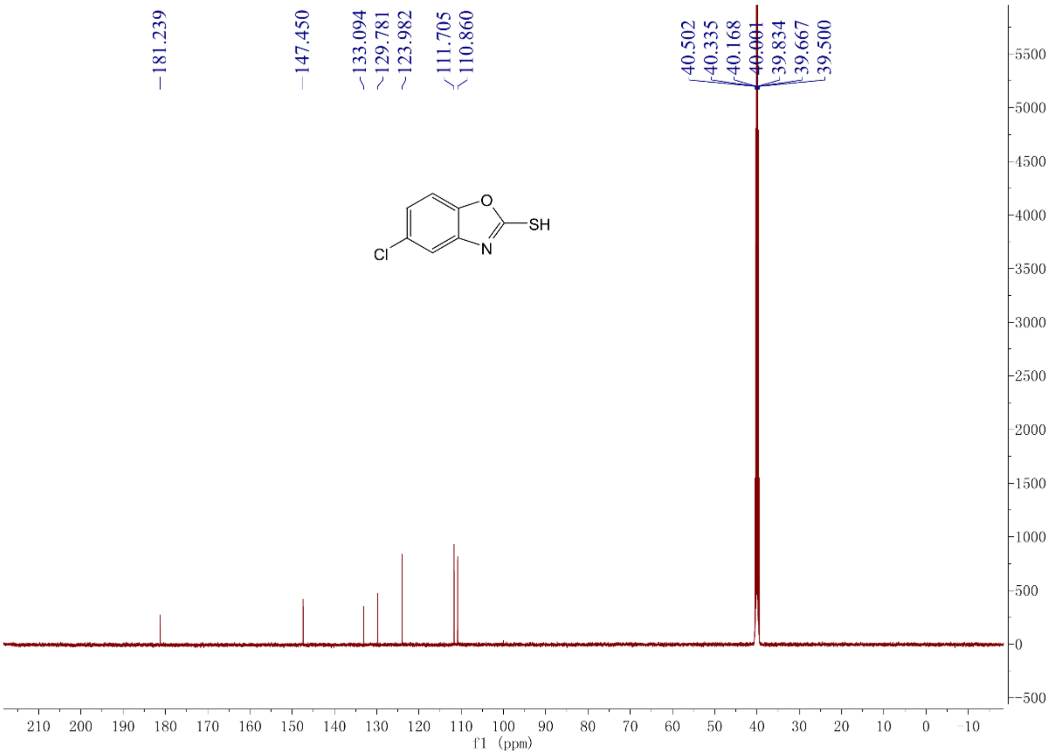

Figure 22: ^{13}C NMR spectrum of compound **3k**
